# Cumulative Scoring Systems and Nomograms for Predicating Survival in Patients With Glioblastomas: A Study Based on Peripheral Inflammatory Markers

**DOI:** 10.3389/fonc.2022.716295

**Published:** 2022-06-01

**Authors:** Chao Yang, Tian Lan, Yi Wang, Wen-Hong Huang, Si-Man Li, Jie Li, Feng-Ping Li, Yi-Rong Li, Ze-Fen Wang, Zhi-Qiang Li

**Affiliations:** ^1^ Department of Neurosurgery, Zhongnan Hospital of Wuhan University, Wuhan, China; ^2^ Department of Physiology, Wuhan University School of Basic Medical Sciences, Wuhan, China; ^3^ Department of Clinical Laboratory, Zhongnan Hospital of Wuhan University, Wuhan, China

**Keywords:** glioblastoma, nomogram, neutrophil-to-lymphocyte ratio, albumin-to-globulin ratio, survival

## Abstract

Inflammation is a hallmark of cancers. The purpose of the present study was to evaluate the prognostic potential of hematological inflammatory markers in glioblastoma multiforme (GBM) patients. The clinical data of 99 patients with lower-grade gliomas and 88 patients with GBMs were retrospectively analyzed. The optimal cutoff values for peripheral markers were determined by X-tile. Kaplan-Meier and Cox proportional hazard regression analyses were performed to identify markers with prognostic significance. Several scoring systems were constructed by combining these prognostic markers. The predictive accuracies of nomograms incorporating these scoring systems were evaluated by Harrell’s concordance index and receiver operating characteristic curve analysis. GBM patients exhibited higher neutrophil counts (p=0.001), neutrophil-to-lymphocyte ratio (NLR) (p<0.001), and platelet-to-lymphocyte ratio (PLR) (p=0.001), as well as lower lymphocyte counts (p=0.023), lymphocyte-to-monocyte ratio (LMR) (p=0.015), and albumin-to-globulin ratio (AGR) (p=0.003) than those with lower-grade gliomas. Multivariate analysis indicated that a high NLR (> 2.0) (Hazard ratio[HR]=2.519, 95% confidence interval (CI): 1.220-5.204, p=0.013), low LMR (< 2.3) (HR=2.268, 95%CI: 1.172-4.386, p=0.015), or low AGR (< 1.7) (HR=2.924, 95%CI: 1.389-6.135, p=0.005) were associated with poor overall survival in GBM patients. The scoring systems of AGR-NLR, AGR-LMR, and LMR-NLR were associated with GBM survival. The nomogram integrating AGR-NLR score had the best efficacy in predicting GBM survival (c-index=0.874). Pretreatment scores of AGR-NLR, AGR-LMR, and LMR-NLR may serve as prognostic factors for GBM patients, and a nomogram integrating AGR-NLR may provide a reliable tool to facilitate personalized preoperative evaluations.

## Introduction

Glioblastoma multiforme (GBM) is the most common type of malignant brain tumor in adults (1). Despite improvements in surgical resection and radiochemotherapy, GBM patients only have a median overall survival of less than 15 months (2). Some prognostic factors for GBM have been identified, of which isocitrate dehydrogenase 1 (IDH1) mutation and O^6^-methylguanine-DNA methyltransferase (MGMT) promoter methylation have been widely used in clinical practice (3). However, such molecular biomarkers are only obtained postoperatively. Therefore, it is necessary to identify preoperative biomarkers for estimating the clinical outcomes of GBM patients before therapy and to assist guiding individualized management.

Inflammation is a hallmark of cancer that plays a crucial role in tumor progression and metastasis (4–6). Several hematological inflammatory markers, which are readily available from routine blood tests, have been considered as prognostic factors in various types of tumors. Some studies have indicated that an elevated neutrophil-to-lymphocyte ratio (NLR) predicts worse outcomes in patients with various tumors, including colorectal cancer (7), breast cancer (8), prostate cancer (9), renal cell carcinoma (10), and gliomas (11, 12). The prognostic value of the NLR in GBM patients has also been described in several studies (13–15). However, a recent study showed that preoperative NLR was not associated with survival in a GBM cohort treated with combined modality therapy (16). One study also reported that a high platelet-to-lymphocyte ratio (PLR) may serve as a predictor of poor prognosis in GBM (17), but results from other studies do not support the prognostic value of PLR for GBM (14, 18, 19). A lower albumin-to-globulin ratio (AGR), a marker of both nutritional and inflammatory status, is associated with poor prognosis in patients with GBM (20) and several other types of tumors (21–24). A recent meta-analysis also demonstrated that a low AGR was a risk factor for unfavorable survival in patients with high-grade gliomas (Grade III and IV) (25). Moreover, the diagnostic significance of these inflammatory markers in glioma patients has also been reported (26, 27).

In most studies, the prognostic values of inflammatory markers have been assessed individually. Recently, several cumulative prognostic scoring systems based on a combination of two or three peripheral biomarkers have been proposed. For example, a cumulative score combining levels of plasma fibrinogen and serum albumin may represent a prognostic predictor for esophageal cancer (28); additionally, a score combining NLR, AGR, and levels of fibrinogen has been reported to be correlated with tumor grade and prognosis in patients with glioma (20). Since each inflammatory marker may influence tumor progression through different mechanisms, a cumulative scoring system provides a more accurate and comprehensive tool for survival prediction. At present, only few studies have investigated cumulative prognostic scoring systems for GBM. Another concern is the clinical applicability and predictive accuracy of the addition of such cumulative prognostic scores into well-identified prognostic variables. Nomograms are commonly used tools in oncology and provide an estimated numerical prognosis for each individual patient by integrating diverse prognostic and determinant variables.

In the present study, we first identified inflammatory markers that were independently associated with survival in GBM patients, and then constructed several scoring systems based on combinations of these markers. The prognostic significance of these cumulative scores was further evaluated and nomogram models were constructed to predict survival probability. Taken together, our findings shed new light on the prognostic value of peripheral inflammatory markers in GBM patients.

## Materials and Methods

### Study Cohort

We retrospectively identified 187 adult patients diagnosed with Grade-II, -III, or -IV gliomas in the Department of Neurosurgery at Zhongnan Hospital of Wuhan University between January 2016 to May 2019. Inclusion criteria were as follows: (1) age ≥ 18 years; (2) histologically confirmed diagnosis; and (3) clinical information and data of preoperative peripheral blood routine tests and liver function tests were available. Exclusion criteria were as follows: (1) patients who received chemotherapy (including corticosteroids) and/or radiotherapy before surgery; (2) patients with a history of other malignant tumors or chronic inflammatory diseases (including autoimmune diseases and infection); (3) patients diagnosed with recurrent gliomas; or (4) patients who died during the perioperative period. All relevant data were treated with confidentiality according to the Declaration of Helsinki (29). The endpoint of follow-ups was August 31, 2020. The blood samples were collected at 5:00 to 6:00 within 1 day after hospitalization. A completed STROBE checklist was shown in supplementary STROBE statement.

### Ethics Approval

This study was approved by the Ethics Committee of Zhongnan Hospital of Wuhan University (No. 2019048).

### Data Collection

Demographic and clinical information were extracted from an electronic medical system and included gender, age at diagnosis, tumor location, tumor grade, the statuses of IDH1 mutation and MGMT promoter methylation (positive, negative, or not determined), preoperative Karnofsky performance status (KPS), the extent of resection [gross total resection (GTR) ≥95%, non-GTR<95%], and postoperative chemotherapy and/or radiotherapy. Data comprising preoperative counts of neutrophils, lymphocytes, and platelets, as well as the concentrations of albumin and globulin, were also collected. Based on these data, the NLR, PLR, AGR, and lymphocyte-to-monocyte ratio (LMR) were calculated. Overall survival (OS) was defined as the interval from the time of surgery to death or the endpoint of follow-up. Follow-up was conducted by telephone.

### Nomograms for Predicting OS in GBM Patients

Based on multivariate analysis, variables with independent prognostic value—including KPS, chemoradiotherapy, the extent of resection, and scoring systems combining inflammatory markers—were integrated to develop nomograms. Each variable was assigned different points according to its weight, which is indicated at the top scale. The total points of all included variables generated numerical predictions of the 0.5-, 1.0 and 1.5-year survival rates, with higher scores indicating worse outcomes.

### Statistical Analysis

Continuous variables are presented as the mean ± standard deviation if they were normally distributed, and are presented as the median and interquartile range (IQR) if they were non-normally distributed. Categorical variables are presented as the number of cases and their corresponding percentages. Continuous variables were analyzed using nonparametric Mann-Whitney U tests or unpaired t-tests, and binary and ordinal categorical variables were analyzed using chi-square tests and the Kruskal-Wallis tests, respectively. The optimal cutoff values for the peripheral markers were identified *via* X-tile software (version 3.6.1, http://medicine.yale.edu/lab/rimm/research/software.aspx), and correlations among the variables were assessed by Spearman correlation coefficients. Survival curves were plotted using the Kaplan-Meier method and were analyzed by the log-rank test. Univariate as well as multivariate Cox proportional hazard regression analyses were conducted to evaluate independent prognostic factors for OS. Nomograms were constructed using the R rms package to predict the survival rates at 0.5, 1.0 and 1.5 years, taking into account all the independent prognostic markers. The discrimination of nomograms was assessed by Harrell’s concordance index (C-index) and time-dependent receiver operating characteristic (ROC) curve analysis. Calibration plots were made to evaluate the consistency between the predicted and observed values. Statistical analysis was performed with SPSS (version 24.0, IBM Corporation, Armonk, NY, USA) or R software (version 4.0.2; Institute for Statistics and Mathematics, Vienna, Austria). A two-tailed P < 0.05 was considered statistically significant.

## Results

### Clinical Characteristics

The clinical characteristics of all patients are presented in **Table 1**. A total of 187 patients (76 females and 111 males) were recruited in this study—including 52 patients with grade-II gliomas, 47 patients with grade-III gliomas, and 88 patients with grade-IV GBMs—with a mean age of 50.3 years (range 21–81 years). Among GBM patients, 69.3% underwent non-GTR, and 56.8% received radiotherapy and/or chemotherapy after surgery. GBM patients harboring an IDH1 R132H mutation or MGMT promoter methylation consisted of 4 (4.5%) and 33 (37.5%) patients, respectively.

**Table 1:** Clinical and pathological characteristics of patients.

Characteristics	All patients	GradeⅡ/Ⅲ	Grade Ⅳ	P Value
Count, n (%)	187 (100)	99 (52.9)	88 (47.1)	
Sex, n (%)				0.944
Female	76 (40.6)	40 (40.4)	36 (40.9)	
Male	111 (59.4)	59 (59.6)	52 (59.1)	
Age (y) (Mean ± SD)	50.3 ± 12.9	45.0 ± 12.0	56.3 ± 11.2	<0.001
Location				0.855
Frontal	55 (29.4)	32 (32.3)	23 (26.1)	
Temporal	41 (21.9)	20 (20.2)	21 (23.9)	
Parietal	17 (9.1)	8 (8.1)	9 (10.2)	
Multiple	36 (19.3)	18 (18.2)	18 (20.5)	
Others	38 (20.3)	21 (21.2)	17 (19.3)	
KPS (Mean ± SD)	78.1 ± 14.9	85.3 ± 9.0	70.2 ± 16.1	<0.001
Radio- and/or chemotherapy, n (%)				<0.001
Yes	129 (69.0)	79 (79.8)	50 (56.8)	
No	51 (27.3)	14 (14.1)	37 (42.0)	
Missing	7 (3.7)	6 (6.1)	1 (1.1)	
Resection, n (%)				<0.001
GTR	81 (43.3)	54 (54.5)	27 (30.7)	
Non-GTR	102 (54.5)	41 (41.4)	61 (69.3)	
Missing	4 (2.1)	4 (4.0)	0 (0)	
IDH1^R132H^, n (%)				<0.001
Yes	59 (31.6)	55 (55.6)	4 (4.5)	
No	119 (63.6)	36 (36.4)	83 (94.3)	
Not done	9 (4.8)	8 (8.1)	1 (1.1)	
MGMT promoter methylation, n (%)				0.001
Yes	91 (48.7)	58 (58.6)	33 (37.5)	
No	79 (42.2)	30 (30.3)	49 (55.7)	
Not done	17 (9.1)	11 (11.1)	6 (6.8)	
WBC (10^9^/L) (Mean ± SD)	6.8 ± 2.5	6.5 ± 2.5	7.1 ± 2.4	0.016
Neutrophil (10^9^/L) (Mean ± SD)	4.5 ± 2.3	4.0 ± 2.3	5.0 ± 2.3	0.001
Lymphocyte (10^9^/L) (Mean ± SD)	1.7 ± 0.9	1.9 ± 1.2	1.5 ± 0.5	0.023
Platelet (10^9^/L) (Mean ± SD)	190.4 ± 55.4	181.8 ± 50.7	200.1 ± 59.1	0.082
Monocyte (10^9^/L) (Mean ± SD)	0.5 ± 0.2	0.5 ± 0.2	0.5 ± 0.2	0.535
Albumin (g/L) (Mean ± SD)	39.9 ± 3.5	40.1 ± 3.7	39.7 ± 3.3	0.417
NLR (Mean ± SD)	3.2 ± 2.4	2.6 ± 2.1	3.8 ± 2.6	<0.001
PLR (Mean ± SD)	130.0 ± 65.5	115.9 ± 57.8	145.8 ± 70.3	0.001
LMR (Mean ± SD)	3.8 ± 1.6	4.0 ± 1.5	3.5 ± 1.7	0.015
AGR (Mean ± SD)	1.6 ± 0.3	1.6 ± 0.3	1.5 ± 0.2	0.003
OS (months) (Mean ± SD)	18.7 ± 12.4	25.1 ± 12.4	12.0 ± 8.2	<0.001

KPS, Karnofsky performance status; GTR, gross total resection; IDH, isocitrate dehydrogenase; MGMT, O^6^-methylguanine-DNA methyltransferase; NLR, neutrophil to lymphocyte ratio; PLR, platelet to lymphocyte ratio; LMR, lymphocyte to monocyte ratio; AGR,albumin to globulin ratio; OS, overall survival.

### Calculation of Sample Size

According to previous published literature (30) and clinical experience, it is expected that the mean value of NLR (μ_A)_ in the lower grade glioma group is 1.99, the mean value of NLR (μ_B)_ in the GBM group is 2.64, and the standard deviation (σ) is 1.5. Bilateral test is adopted. Sample size was calculated according to the following formula: ${\text{n}}_{\text{B}}=\left(1+\frac{1}{\text{κ}}\right){\left(\sigma \frac{z_{1-\frac{\alpha }{2}}+z_{1-\beta }}{\text{μA}-\text{μB}}\right)}^{2}$ (31). The following parameters were used: κ=1, class I error α =0.05, z _1-α / 2_ = 1.96, class II error β=0.2, z _1 – β_ = 0.84. The effect variable (D-Cohen) was 0.43. According to the calculation, lower grade glioma and GBM groups requires 84 cases in each group. In our study, 99 cases of lower grade glioma and 88 cases of GBM were collected, meeting the research requirements.

### Comparison of Peripheral Inflammatory Markers Between Lower-Grade Glioma (LGG) and GBM

Differences in neutrophil, lymphocyte, platelet, and monocyte counts—as well as NLR, PLR, LMR, and AGR—between patients with LGG and GBM were compared using Mann-Whitney U tests, and the difference in serum albumin levels was analyzed using an unpaired t-test. Compared to the parameters in LGG patients, GBM patients exhibited higher neutrophil counts, NLR, and PLR, as well as lower lymphocyte counts, LMR, and AGR. The absolute counts of platelets and monocytes, as well as serum albumin levels, were comparable between the two groups (**Table 1**). Besides, ROC curve and the corresponding area under the curve (AUC) value were used to assess the diagnostic efficacy in distinguishing GBM from LGG. Among all the single inflammatory markers, NLR showed the best diagnostic value for GBM versus LGG with AUC of 0.665 (0.586-0.743). We also combined these hematological markers in order to increase the diagnostic significance, of which AGR+NLR exhibited the best accuracy for GBM diagnosis with the AUC value of 0.706 (0.630-0.782) in common with the combination of LMR, NLR and AGR (**Supplemental Figure S1**).

### Association Between Inflammatory Markers and Survival in GBM Patients

**Figure 1:**
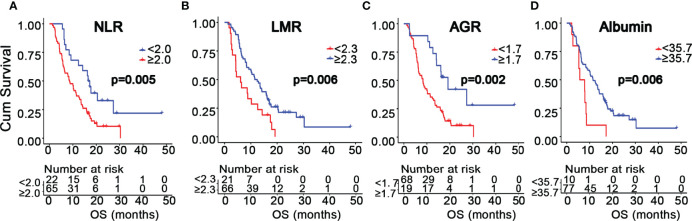
Kaplan-Meier survival curves of GBM patients based on the cutoff values of NLR **(A)**, LMR **(B)**, AGR **(C)**, and albumin **(D)**.

Since all of these peripheral markers were determined to be continuous variables, the optimal cutoff value for each marker was determined using X-tile software. As shown in **Supplementary Figure S2**, the cutoff values for neutrophil, lymphocyte, platelet, and albumin levels were 4.7 (10^9^ cells/L), 2.3 (10^9^ cells/L), 208 (10^9^ cells/L), and 35.7 g/L, respectively; additionally, those of NLR, PLR, LMR, and AGR were 2.0, 213.0, 2.3, and 1.7, respectively. Next, GBM patients were divided into two subgroups according to the cutoff value of each marker. We examined whether GBM patients in these subgroups had different OS values. Patients with high neutrophil levels and NLR or low lymphocyte, albumin, LMR, and AGR levels exhibited shorter OS (**Supplementary Table S1**). Next, we evaluated the prognostic value of these markers in GBM patients. Kaplan-Meier survival analysis demonstrated that GBM patients with a high NLR (p = 0.005) had a worse OS, whereas those with a high LMR (p = 0.006), AGR (p = 0.002), or albumin (p = 0.006) had a better OS (**Figure 1**). There was no significant association between neutrophil (p = 0.057), lymphocyte (p = 0.224), platelet (p = 0.311), or PLR (p = 0.290) values with OS (**Supplementary Figure S3**). Both univariate and multivariate analysis revealed that NLR, LMR, and AGR were significantly associated with survival, in addition to KPS, postoperative radiochemotherapy, and the extent of tumor resection (**Table 2**). Moreover, Spearman correlation analysis showed that there was no correlation between NLR or LMR with AGR, and only a weak correlation between NLR and LMR (r = −0.613, p < 0.01) (**Supplementary Figure S4**). Unexpectedly, our analysis did not show a significant association of MGMT promoter methylation or IDH1 mutation with OS (**Supplementary Figure S5**). Univariate analysis showed that the serum albumin level was a significant variable associated with OS, but multivariate analysis did not corroborate this finding.

**Table 2:** Univariate and multivariate analyses of OS in GBM cohorts.

Variables	Univariate Analysis		Multivariate analysis	
	HR (95% CI)	P value	HR (95% CI)	P value
Sex (female/male)	1.174 (0.729-1.891)	0.509	/	/
Age (<60/≥60)	0.684 (0.428-1.092)	0.111	/	/
Location	1.097 (0.935-1.287)	0.258	/	/
KPS (<60/≥60)	13.422(6.618-27.223)	<0.001	6.117(2.795-13.388)	<0.001
Radio- and/or chemotherapy (no/yes)	9.098 (4.874-16.982)	<0.001	3.539 (1.683-7.443)	0.001
Resection (GTR/non-GTR)	4.096 (2.303-7.286)	<0.001	2.573 (1.321-5.012)	0.005
IDH1^R132H^ (yes/no)	1.671 (0.525-5.322)	0.385	1.079 (0.320-3.641)	0.902
MGMT promoter methylation(yes/no)	1.655 (0.991-2.763)	0.054	1.631 (0.928-2.865)	0.089
Neutrophil (≥4.7/<4.7) (10^9^/L)	1.564 (0.982-2.491)	0.059	1.001 (0.551-1.818)	0.998
Lymphocyte (≥2.3/<2.3) (10^9^/L)	0.539 (0.195-1.486)	0.232	/	/
Platelet (≥208/<208) (10^9^/L)	1.284 (0.790-2.086)	0.313	/	/
Albumin (<35.7/≥35.7) (g/L)	2.545 (1.278-5.070)	0.008	1.585 (0.672-3.738)	0.292
NLR (≥2.0/<2.0)	2.217 (1.247-3.942)	0.007	2.519 (1.220-5.204)	0.013
PLR (≥213.0/<213.0)	1.381 (0.756-2.524)	0.294	/	/
LMR (<2.3/≥2.3)	2.062 (1.221-3.484)	0.007	2.268 (1.172-4.386)	0.015
AGR (<1.7/≥1.7)	2.632 (1.377-5.025)	0.003	2.924 (1.389-6.135)	0.005

KPS, Karnofsky performance status; GTR, gross total resection; IDH, isocitrate dehydrogenase; MGMT, O^6^-methylguanine-DNA methyltransferase; NLR, neutrophil to lymphocyte ratio; PLR, platelet to lymphocyte ratio; LMR, lymphocyte to monocyte ratio; AGR, albumin to globulin ratio; HR, hazard ratio; CI, confidence interval.

### Association Between Scoring Systems of Combined Inflammatory Markers and Survival in GBM Patients

**Figure 2:**
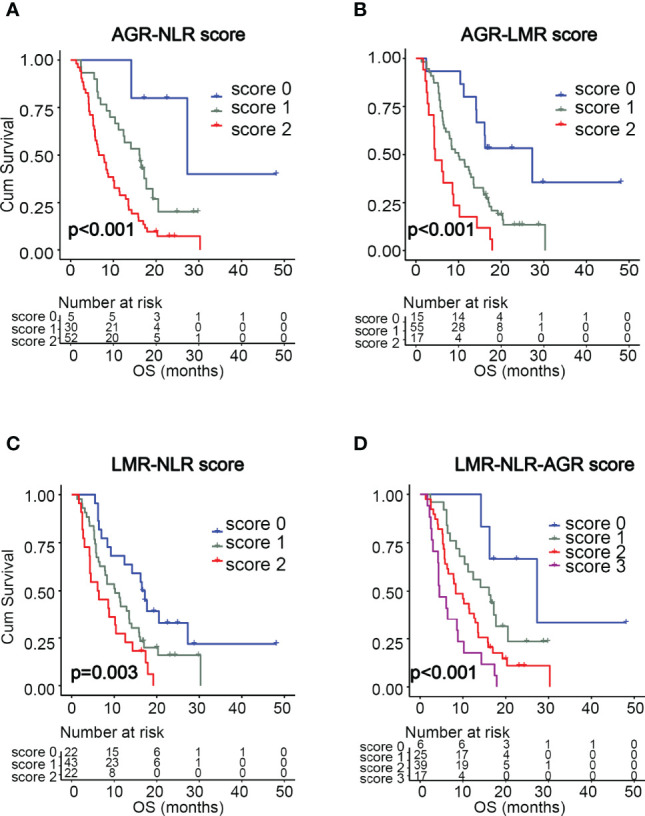
Kaplan-Meier survival curves of GBM patients based on the scoring systems of AGR-NLR **(A)**, AGR-LMR **(B)**, LMR-NLR **(C)**, and LMR-NLR-AGR **(D)**.

We found that NLR, LMR, and AGR were significantly associated with survival. Next, we investigated whether combinations of these inflammatory markers could serve as more powerful predictors of GBM survival. As shown in **Table 3**, four prognostic scoring systems were constructed by incorporating any two or all of the three variables, including AGR-NLR, AGR-LMR, LMR-NLR, and LMR-NLR-AGR scoring systems. The scores in these systems were determined by the presence or absence of the each variable’s status associated with unfavorable survival (i.e. high NLR [>2.0], low LMR [<2.3], or low AGR [<1.7]). For AGR-NLR, AGR-LMR, and LMR-NLR scores, the systems were classified into three groups: a score of 0 (neither of the two variables were present); score of 1 (either of the variables was present); and a score of 2 (both variables were present). The LMR-NLR-AGR scoring system was classified into four groups: a score of 0 (none of the three variables were present); a score of 1 (any one of the three variables was present); a score of 2 (any two of the variables were present); and a score of 3 (all three variables were present). The OS data for each scoring system are shown in **Table 3**. Kaplan-Meier survival analysis showed that AGR-NLR, AGR-LMR, LMR-NLR, and LMR-NLR-AGR scores were significantly associated with OS (p < 0.001, p < 0.001, p = 0.003, and p < 0.001, respectively, **Figure 2**). As shown in **Table 4**, results of multivariate analysis demonstrated that—for AGR-LMR and LMR-NLR scoring systems—both a score of 1 and a score of 2 were associated with an unfavorable OS. For the AGR-NLR scoring system, only a score of 2 was associated with a worse OS. However, neither of the scores in the LMR-NLR-AGR system was predictive factor for OS in GBM cohort. In this study, 69.3% of GBM patients received non-gross total resection. We further assessed the prognostic significance of these scoring system for patients with non-gross total resection. Similar results were observed with AGR-NLR, AGR-LMR and LMR-NLR scoring system (**Supplementary Table S2**). Moreover, the scores of LMR-NLR-AGR system were also associated with a worse OS for patients with non-gross total resection (**Supplementary Table S2**). Due to the small size of GBM patients with gross-total resection, we did not perform further analysis in this subpopulation.

**Table 3:** Overall survival of GBM cohorts based on different score systems.

Score		N (%)	Overall survival		P value
Variables	Definition		Mean ± SD	Median ± IQR	
**AGR-NLR^a^**					
Score 0	AGR>1.7 and NLR<2.0	5 (5.7)	25.9 ± 13.4	22.5 (22.0)	reference
Score 1	AGR<1.7 or NLR>2.0	30 (34.1)	14.1 ± 7.0	16.2 (9.3)	0.233
Score 2	AGR<1.7 and NLR>2.0	52 (59.1)	9.3 ± 6.6	7.2 (9.2)	0.003
**AGR-LMR^b^**					
Score 0	AGR>1.7 and LMR>2.3	15 (17.0)	18.7 ± 10.5	16.7 (8.4)	reference
Score 1	AGR<1.7 or LMR<2.3	55 (62.5)	11.6 ± 7.1	10.0 (11.0)	0.049
Score 2	AGR<1.7 and LMR<2.3	17 (19.3)	7.0 ± 5.2	4.5 (6.7)	<0.001
**LMR-NLR^c^**					
Score 0	LMR>2.3 and NLR<2.0	23 (26.1)	16.7 ± 9.9	16.6 (13.0)	reference
Score 1	LMR<2.3 or NLR>2.0	43 (48.9)	11.4 ± 7.2	10.2 (10.6)	0.044
Score 2	LMR<2.3 and NLR>2.0	22 (25.0)	8.2 ± 5.8	6.3 (10.1)	0.001
**LMR-NLR-AGR^d^**					
Score 0	LMR>2.3 and NLR<2.0 and AGR>1.7	6 (6.8)	24.3 ± 12.6	19.9 (16.8)	reference
Score 1	LMR<2.3 or NLR>2.0 or AGR<1.7	25 (28.4)	14.3 ± 7.2	16.1 (9.8)	0.627
Score 2	LMR<2.3 and NLR>2.0, or LMR<2.3 and AGR<1.7, or NLR>2.0 and AGR<1.7	39 (44.3)	10.7 ± 6.9	8.3 (10.5)	0.022
Score 3	LMR<2.3 and NLR>2.0 and AGR<1.7	17 (19.3)	7.0 ± 5·2	4.5 (6·7)	0.001

^a,b,c,d^: the data was not available for one case in each score system.

NLR, neutrophil to lymphocyte ratio; PLR, platelet to lymphocyte ratio; LMR, lymphocyte to monocyte ratio; AGR, albumin to globulin ratio; SD, standard deviation; IQR, interquartile range.

**Table 4:** Univariate and multivariate analyses for OS in GBM cohorts based on score systems.

Score system	Univariate Analysis		Multivariate analysis	
	HR (95% CI)	P value	HR (95% CI)	P value
**AGR-NLR**		0.001		<0.001
Score 0	1 (reference)		1 (reference)	
Score 1	3.106 (0.720-13.401)	0.129	1.582 (0.313-7.991)	0.579
Score 2	6.948 (1.666-28.968)	0.008	4.908 (1.031-23.368)	0.046
**AGR-LMR**		<0.001		0.003
Score 0	1 (reference)		1 (reference)	
Score 1	2.587 (1.215-5.511)	0.014	2.884 (1.228-6.772)	0.015
Score 2	5.650 (2.390-13.359)	<0.001	5.262 (2.027-13.661)	0.001
**LMR-NLR**		0.001		0.032
Score 0	1 (reference)		1 (reference)	
Score 1	1.912 (1.042-3.511)	0.036	2.360 (1.122-4.966)	0.024
Score 2	3.140 (1.595-6.181)	0.001	3.136 (1.283-7.669)	0.012
**LMR-NLR-AGR**		<0.001		0.001
Score 0	1 (reference)		1 (reference)	
Score 1	2.290 (0.664-7.899)	0.190	0.826 (0.195-3.502)	0.796
Score 2	4.298 (1.306-14.147)	0.016	2.565 (0.660-9.966)	0.174
Score 3	8.034 (2.294-28.135)	0.001	4.096 (0.965-17.382)	0.056

NLR, neutrophil to lymphocyte ratio; LMR, lymphocyte to monocyte ratio; AGR, albumin to globulin ratio; HR, hazard ratio; CI, confidence interval.

### Nomograms for Predicting OS in GBM Patients

Based on the results of multivariate analysis, several independent prognostic factors were identified, including KPS, chemoradiotherapy, resection, AGR-NLR score, AGR-LMR score, and LMR-NLR score. Then, three nomograms were established to evaluate the value of these variables to predict the 0.5-, 1.0- and 1.5-year OS in GBM patients.

**Figure 3:**
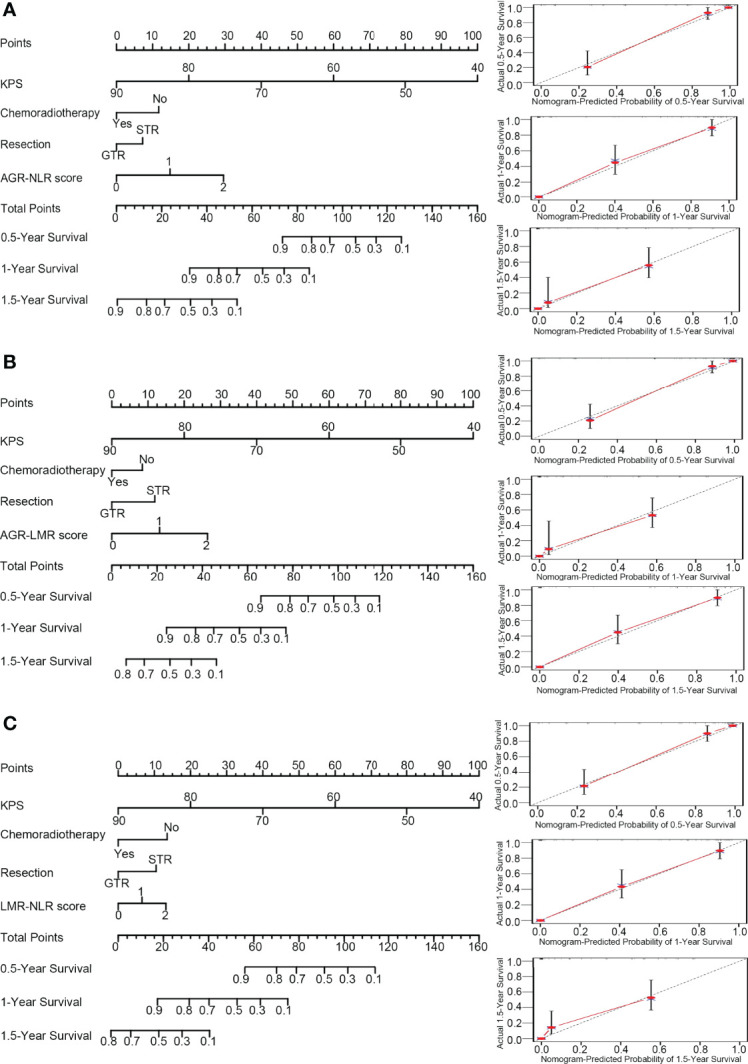
Nomograms and calibration curve. In the nomograms (left panel), each variable was assigned different points that are indicated at the top scale, and the total points of all variables generated a numerical prediction of the 0.5-, 1.0- and 1.5-year survival rates, where a higher score indicates a worse outcome. In the calibration curve (right panel), the dotted line indicates the ideal prediction and the full red line indicates the performance of the nomogram. **(A)** Nomogram and calibration curve concerning AGN-NLR score; **(B)** Nomogram and calibration curve concerning AGR-LMR score; **(C)** Nomogram and calibration curve concerning LMR-NLR score.

In the nomogram incorporating AGR-NLR, KPS contributed most to the prognosis, followed by AGR-NLR score, chemoradiotherapy, and resection. This nomogram showed a good accuracy for predicting the survival rate of GBM patients, with a c-index of 0.874. The bootstrapped calibration plot of the nomogram predicting 0.5-, 1.0-, and 1.5-year OS performed well with the ideal model (**Figure 3A**), indicating the validation of this nomogram.

In the nomogram incorporating AGR-LMR, KPS contributed most to the prognosis, followed by AGR-LMR score, resection, and chemoradiotherapy. The c-index was 0.867, indicating its high accuracy for predicting OS. The calibration curve also indicated the strong predictive ability of this nomogram (**Figure 3B**).

In the nomogram incorporating LMR-NLR, KPS also contributed most to the prognosis, and chemoradiotherapy and LMR-NLR score had a similar weight on the risk score, followed by resection. The c-index was 0.866. The validation of this nomogram was also demonstrated by a well-fitted calibration curve (**Figure 3C**).

Of the three nomograms, the one incorporating AGR-NLR had the highest c-index, showing a minor advantage of accuracy to predict OS. Consistently, time-dependent ROC analysis also indicated that the nomogram incorporating AGR-NLR had the highest area under the curve (AUC) value to predict OS at 0.5, 1.0 or 1.5 years (0.703, 0.708, and 0.738, respectively) (**Supplementary Figure S6**).

## Discussion

Accumulating evidence has identified a crucial role of inflammation in tumor biology (32). It is widely accepted that the initiation, progression, and metastasis of many types of tumors are closely correlated with local and/or systemic inflammation (5). Peripheral inflammation and nutritional status have been reported to correlate with OS in cancer patients, including those with gliomas (13, 15, 21, 33, 34). In the present study, we found that, compared to parameters in LGG patients, GBM patients exhibited higher neutrophil counts, NLR, and PLR, as well as lower lymphocyte counts, LMR, and AGR. Of these variables, a high NLR, low LMR, and low AGR were associated with unfavorable OS in GBM patients. Furthermore, we found that cumulative scores of AGR-NLR, AGR-LMR, and LMR-NLR may serve as predictors of GBM survival, and a nomogram integrating AGR-NLR had the best predictive accuracy and discrimination for estimating OS. Moreover, AGR-NLR score exhibited the best accuracy for GBM diagnosis, which is consistent with a previous study (35).

Patients with malignant tumors often endure a status of neutrophilia and relative lymphopenia that is predominantly due to the overproduction of tumor-cell-derived granulocyte colony stimulating factor (G-CSF), which has the potential to divert bone marrow hematopoiesis away from the lymphocytic lineage toward the granulocytic lineage (36, 37). In line with these studies, our present study also found that GBM patients had a higher neutrophil count and lower lymphocyte count compared to those of LGG patients. In contrast, there was no significant difference in platelet or monocyte counts. These changes in blood cell counts contributed to a higher NLR and PLR, as well as a lower LMR, in GBM patients. Multivariate analysis indicated that high NLR (> 2.0) and low LMR (< 2.3) were poor prognostic indicators for GBM patients, but high PLR (> 213.0) was not. A cutoff value of 4 for preoperative NLR has most commonly been used in GBM studies and has been shown to be associated with glioma grade and poor survival in GBM patients (13–15). Our present data indicated that NLR at a lower cutoff value may serve as a potential predictor for GBM survival.

The mechanisms underlying the prognostic impact of NLR and LMR have not been fully clarified. Neutrophils and lymphocytes are the main cell types mediating inflammatory and immune responses. Decreased lymphocyte counts in the blood and tumor stroma lead to suppression of immune responses against tumors. Tumor-derived cytokines, such as tumor growth factor β (TGF-β), induce the activation of neutrophils with a pro-tumoral phenotype (38). Activated neutrophils can produce and secrete various molecules, such as vascular endothelial growth factor (39) and matrix metalloproteinases (40), to promote tumor progression, metastasis, and angiogenesis. Moreover, neutrophils have the ability to inhibit cytotoxic activities of T cells and lymphokine-activated killer cells (41, 42). After recruitment into tumor tissue, monocytes differentiate into tumor-associated macrophages, which have pro-tumor functions and are closely related to tumor progression (43). Furthermore, peripheral monocyte counts may reflect the formation or presence of tumor-associated macrophages. Therefore, both high NLR and low LMR in patients with cancer indicate an immunosuppressive and pro-tumor status. Interestingly, one study showed that an elevated LMR also predicted a better response to chemotherapy in patients with breast cancer (44).

The AGR reflects a comprehensive status of nutrition and inflammation. Malnutrition is common in patients with cancer. Accumulating evidence has indicated that a status of malnutrition predicts a worse OS in many cancers (45). Albumin, a major component of serum proteins, is widely considered as an indicator of nutritional status (46). Inconsistent with a previous study (47), we did not find a significant association between preoperative serum albumin counts and OS in GBM patients in our present study. In accordance with our present results, a recent meta-analysis also reported that an elevated AGR significantly correlated with a pro-longed survival in GBM patients (25). A relatively high albumin level may indicate a satisfactory tolerance to chemoradiotherapy and a better quality of life postoperatively. Globulin is a large family consisting of immunoglobulins, C-reactive protein (CRP), and complements. Since these proteins are induced in a state of inflammation, an elevated serum globulin level indicates the presence of systemic inflammation (48). Therefore, a low AGR may indicate not only malnutrition and an inadequate antitumor immunity state, but may also indicate the presence of cancer-related inflammation (20).

Since NLR, LMR, and AGR may influence tumor progression through different mechanisms, a combination of these markers may provide a more comprehensive tool for survival prediction. Thus, in our present study, we designed a series of cumulative prognostic scoring systems based on different combinations of NLR, LMR, and AGR. For AGR-LMR and LMR-NLR scoring systems, patients with either of these risk predictors (score 1) or both of them (score 2) had worse clinical outcomes than those with neither of them. For the AGR-NLR scoring system, the presence of both risk predictors was associated with an unfavorable OS. The scoring system of NLR-LMR-AGR was unable to serve as an independent predictor of OS in GBM cohort. However, the scores of NLR-LMR-AGR system were associated with outcomes of the subpopulation receiving non-gross total resection. This subgroup analysis was not performed in patients receiving gross total resection due to the very small size. The inconsistent results between whole GBM cohort and subpopulation with non-gross total resection indicated that the extent of resection may have effect on the prognostic significance of NLR-LMR-AGR system. Therefore, NLR-LMR-AGR system may be not a good independent predictor for GBM patients. A large sample size study is needed to further clarify the correlation. A previous study showed that a scoring system combining fibrinogen, NLR, and AGR may serve as an independent prognostic factor in glioma patients (20). In this previous study, some well-defined molecular markers related to survival—such as IDH1 and MGMT methylation status—were not included in survival analysis. These molecular markers were incorporated in our present study but did not show any prognostic significance. This is most likely due to the small population of our GBM cohort and rarely few cases with IDH1 mutations. Although many studies have reported that GBM patients with MGMT methylation have longer survival than those without MGMT methylation, our recent meta-analysis indicated that the survival benefit from MGMT methylation was closely associated with temozolomide therapy (49).

Nomograms are novel and visual calculating scale models that have been widely used in oncology research (50), as they usefully provide a numerical estimation for a clinical event in an individual patient. Based on multivariate analysis, independent prognostic factors including KPS, chemoradiotherapy, and the extent of resection were included as variables for nomograms in our present study. AGR-NLR, AGR-LMR, and LMR-NLR scores were also incorporated into nomograms. Of these variables, KPS showed the highest risk score. The nomogram incorporating AGR-NLR score had the highest c-index and AUC value of the time-dependent ROC curve analysis, indicating the best accuracy for prognostic prediction. Therefore, a nomogram integrating KPS, chemoradiotherapy, extent of resection, and AGR-NLR score is primarily recommended to predict prognosis for GBM patients. The score system in our study may be used to stratify patients according to their prognostic risk, and high-risk patients may opt for closer follow-up evaluation and more aggressive subsequent treatment. It is noteworthy that this proposed nomogram provides a preoperative tool for clinicians and health administrators to predict survival probability, rather than directly offering criteria for therapeutic decisions. Therefore, decision curve analysis (DCA), a method for evaluating the clinical benefits of an alternative prediction model or diagnostic test, was not applied to the current prognostic nomogram models (51).

There are also some limitations of our present study. First, the retrospective nature of our study may have led to selection bias. Second, the optimal cutoff values for these inflammatory markers have not been defined or recommended. Importantly, different cutoff values may be a contributor to the inconsistent results observed across different studies. Third, a relatively small size of GBM patients was included in our present study, and there were not enough patients harboring IDH1 mutations to perform subgroup analysis. Therefore, the importance and significance of IDH1 mutations and MGMT methylation in nomogram analysis may have been underestimated in our present study. Hence, future large-scale, multi-center, and prospective studies are needed to clarify these concerns.

## Conclusion

In addition to preoperative NLR, LMR, and AGR, the cumulative scores of AGR-NLR, AGR-LMR, and LMR-NLR may serve as prognostic predictors for GBM patients. A nomogram model integrating the AGR-NLR may provide a reliable tool to facilitate a personalized preoperative evaluation of survival. To further facilitate the clinical utility of nomograms, free online software for calculating the survival probability of GBM patients has been provided (https://yangchao.shinyapps.io/dynnomapp/).

## Data Availability Statement

The raw data supporting the conclusions of this article will be made available by the authors, without undue reservation.

## Ethics Statement

The studies involving human participants were reviewed and approved by Ethics Committee of Zhongnan Hospital of Wuhan University (No. 2019048). The patients/participants provided their written informed consent to participate in this study. Written informed consent was obtained from the individual(s) for the publication of any potentially identifiable images or data included in this article.

## Author Contributions

Z-QL conceived and designed the study. CY, TL, YW, and JL analyzed the data and interpreted results. CY, W-HH, S-ML, and F-PL collected epidemiological and clinical data. Y-RL contributed to data interpretation. CY drafted the manuscript; Z-FW and Z-QL revised the manuscript. All authors read and approved the final version of the manuscript.

## Funding

This study was funded by Translational Medicine and Interdisciplinary Research Joint Fund of Zhongnan Hospital of Wuhan University (No. ZNLH201901) and Medical Science Advancement Program of Wuhan University (No. TFJC2018003).

## Conflict of Interest

The authors declare that the research was conducted in the absence of any commercial or financial relationships that could be construed as a potential conflict of interest.

## Publisher’s Note

All claims expressed in this article are solely those of the authors and do not necessarily represent those of their affiliated organizations, or those of the publisher, the editors and the reviewers. Any product that may be evaluated in this article, or claim that may be made by its manufacturer, is not guaranteed or endorsed by the publisher.
