# Supplementary material for: Cumulative Scoring Systems and Nomograms for Predicating Survival in Patients With Glioblastomas: A Study Based on Peripheral Inflammatory Markers
Source: Front Oncol. 2022 Jun 1;12:716295. doi: 10.3389/fonc.2022.716295 (PMC9198436; doi:10.3389/fonc.2022.716295)
Supplement: Supplementary file 1 [file DataSheet_1.doc]

**Supplemental Table S1 Overall survival in GBM cohorts classified by cutoff value of the peripheral markers**

| **Variables** | **OS(months)** | | **P value** |
| --- | --- | --- | --- |
|  | **Mean ± SD** | **Median (IQR)** |  |
| Neutrophil (10^9^/L) |  |  | 0.010 |
| <4.7 | 13.6 ± 7.5 | 14.1 (11.1) |  |
| ≥4.7 | 10.2 ± 8.7 | 8.3 (9.7) |  |
| Lymphocyte (10^9^/L) |  |  | 0.034 |
| <2.3 | 11.5 ± 8.1 | 10.1 (11.1) |  |
| ≥2.3 | 18.3 ± 7.5 | 17.7 (12.8) |  |
| Platelet (10^9^/L) |  |  | 0.064 |
| <208 | 13.2 ± 8.6 | 13.2 (10.9) |  |
| ≥208 | 9.9 ± 7.2 | 7.0 (9.6) |  |
| Albumin (g/L) |  |  | 0.044 |
| <35.7 | 7.1 ± 4.2 | 6.8 (3.8) |  |
| ≥35.7 | 12.6 ± 8.4 | 11.5 (11.2) |  |
| NLR |  |  | 0.002 |
| <2.0 | 16.7 ± 9.9 | 16.6 (13.0) |  |
| ≥2.0 | 10.3 ± 6.9 | 8.6 (11.5) |  |
| PLR |  |  | 0.131 |
| <213.0 | 12·4 ± 8.2 | 11.3 (11.0) |  |
| ≥213.0 | 9·7 ± 8.4 | 6.6 (13.6) |  |
| LMR |  |  | 0.009 |
| <2.3 | 8.1 ± 5.9 | 6.1 (10.6) |  |
| ≥2.3 | 13.2 ± 8.5 | 12.0 (10.9) |  |
| AGR |  |  | 0.004 |
| <1.7 | 10.4 ± 7.0 | 8.3 (10.6) |  |
| ≥1.7 | 17.4 ± 10.0 | 16.4 (6.6) |  |

Abbreviations: NLR, neutrophil to lymphocyte ratio; PLR, platelet to lymphocyte ratio; LMR, lymphocyte to monocyte ratio; AGR, albumin to globulin ratio; SD, standard deviation; IQR, interquartile range.

**Supplemental Table S2 Univariate and multivariate analyses for OS in Non-GTR GBM cohorts based on score systems**

| **Score system** | **Univariate Analysis** | | **Multivariate analysis** | |
| --- | --- | --- | --- | --- |
|  | **HR (95% CI)** | **P value** | **HR (95% CI)** | **P value** |
| **AGR-NLR** |  | 0.016 |  | <0.001 |
| Score 0^a^ | - | - | - | - |
| Score 1 | 1 (reference) |  | 1 (reference) |  |
| Score 2 | 2.084(1.107-3.923) | 0.023 | 4.765(2.061-11.015) | <0.001 |
| **AGR-LMR** |  | 0.016 |  | 0.006 |
| Score 0 | 1 (reference) |  | 1 (reference) |  |
| Score 1 | 2.066(0.727-5.872) | 0.174 | 5.918 (1.685-20.789) | 0.006 |
| Score 2 | 4.251(1.382-13.076) | 0.012 | 8.183(2.248-29.785) | 0.001 |
| **LMR-NLR** |  | 0.048 |  | 0.098 |
| Score 0 | 1 (reference) |  | 1 (reference) |  |
| Score 1 | 1.441(0.628-3.308) | 0.389 | 3.131(0.980-10.002) | 0.054 |
| Score 2 | 2.670(1.092-6.528) | 0.031 | 4.372(1.128-16.945) | 0.033 |
| **LMR-NLR-AGR** |  | 0.009 |  | <0.001 |
| Score 0 | 1 (reference) |  | 1 (reference) |  |
| Score 1 | 1.933(0.955-3.909) | 0.067 | 5.677(2.215-14.552) | <0.001 |
| Score 2 | 3.522(1.581-7.846) | 0.002 | 7.126(2.491-20.392) | <0.001 |
| Score 3^b^ | - | - | - | - |

a: no patients got score 0 in AGR-NLR system.

b: no patients got score 3 in LMR-NLR-AGR system

Abbreviations: NLR, neutrophil to lymphocyte ratio; LMR, lymphocyte to monocyte ratio; AGR, albumin to globulin ratio; HR, hazard ratio; CI, confidence interval.
