# Supplementary material for: Cumulative Scoring Systems and Nomograms for Predicating Survival in Patients With Glioblastomas: A Study Based on Peripheral Inflammatory Markers
Source: Front Oncol. 2022 Jun 1;12:716295. doi: 10.3389/fonc.2022.716295 (PMC9198436; doi:10.3389/fonc.2022.716295)

Supplemental Figure S1. ROC and AUC value of peripheral inflammatory markers of NLR, PLR, LMR, AGR and score system of AGR-NLR, AGR-LMR, LMR-NLR and LMR-NLR-AGR.

A

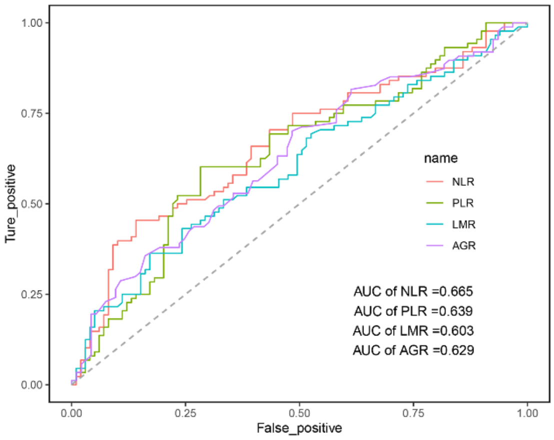

B

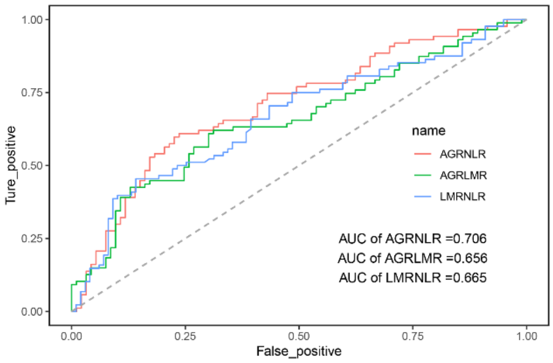

C

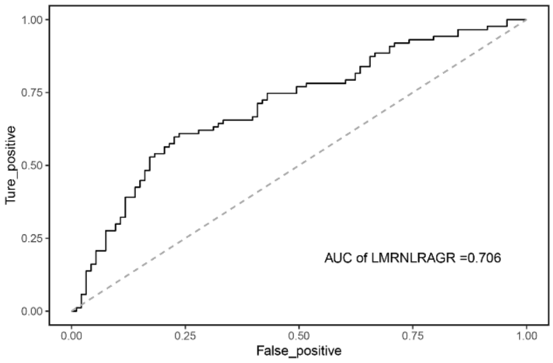

Supplemental Figure S2. Cutoff values of peripheral markers in GBM patients. Neutrophil, lymphocyte, platelet: 109 cells/L; albumin: g/L.

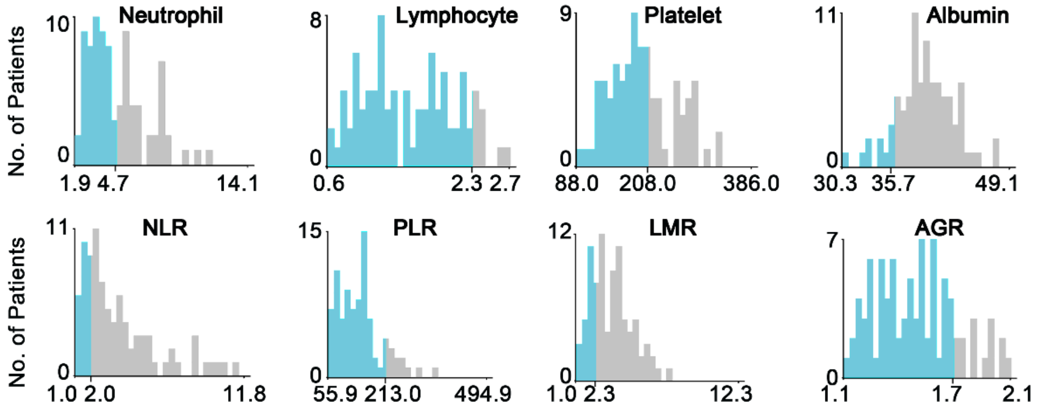

Supplemental Figure S3. Kaplan-Meier survival curves of GBM patients based on the cutoff values of neutrophil, lymphocyte, platelet, and PLR values.

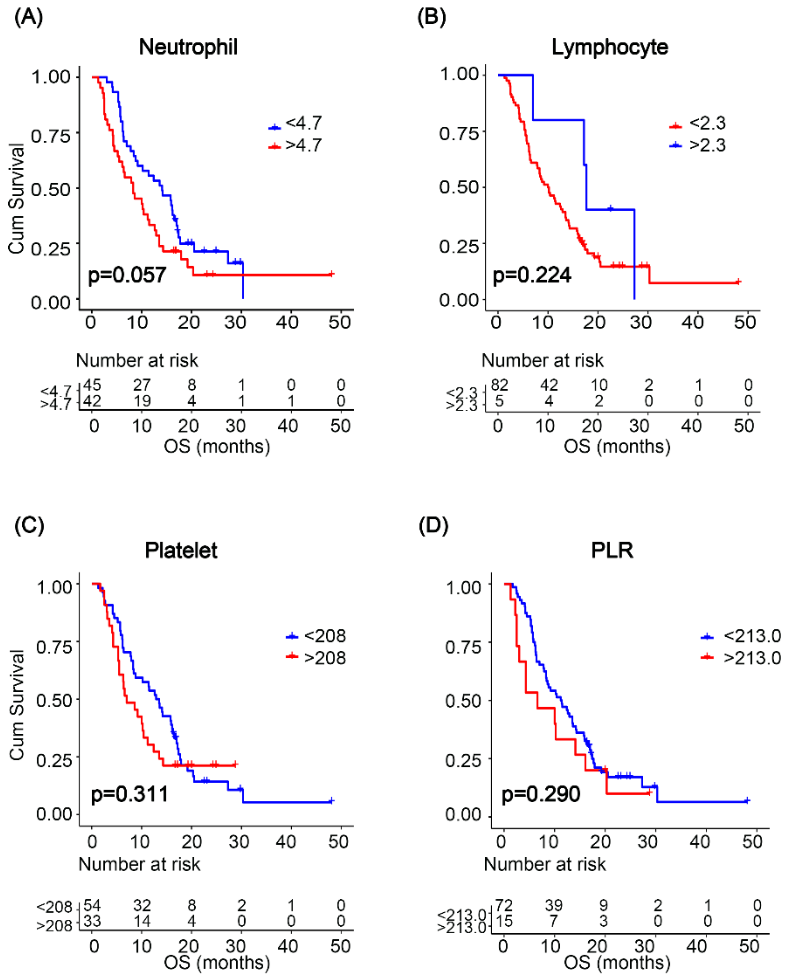

Supplemental Figure S4. Correlation analysis among NLR, LMR, and AGR.

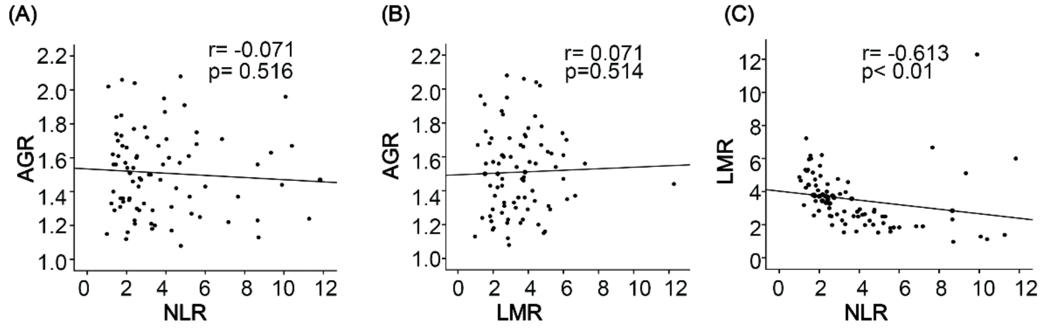

Supplemental Figure S5. Kaplan-Meier survival curves of GBM patients based on the IDH 1 and MGMT status.

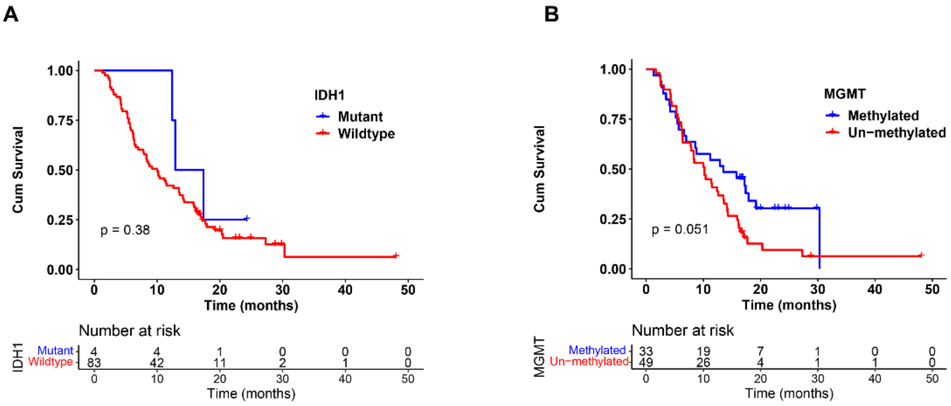

Supplemental Figure S6. Time-dependent ROC curve analysis of three prognostic scoring models for predicting OS in GBM patients.

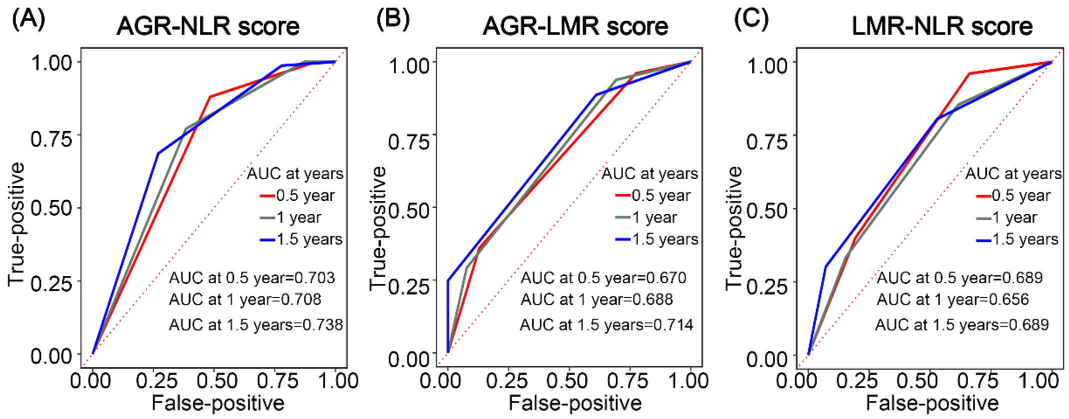

Supplement: Supplementary file 2 [file DataSheet_2.pdf]
